# Consider the woodpecker: The contested more-than-human ethics of biomimetic technology and traumatic brain injury

**DOI:** 10.1177/03063127211052513

**Published:** 2021-10-18

**Authors:** Gregory Hollin

**Affiliations:** University of Leeds, Leeds, UK

**Keywords:** animal models, biomimicry, chronic traumatic encephalopathy, concussion, entanglement, epistemological scaffolding, sport

## Abstract

Chronic Traumatic Encephalopathy, or CTE, is a neurodegenerative disease caused by traumatic brain injury and most frequently associated with contact sports such as American Football. Perhaps surprisingly, the woodpecker – an animal apparently immune to the effects of head impacts – has increasingly figured into debates surrounding CTE. On the one hand, the woodpecker is described as being contra-human and used to underscore the radical inappropriateness of humans playing football. On the other, there have been attempts to mitigate against the risk of CTE through the creation of biomimetic technologies inspired by woodpeckers. In this article I examine the highly politicized encounters between humans and woodpeckers and discuss how the politics of re-/dis-/en-tanglement during these interspecies relations is rendered meaningful. I show here, first, that those who seek to keep the human and the woodpecker apart envisage social overhaul while biomimetic technologies are put to work for the status quo. Second, I stress that different forms of entanglement have diverse sociopolitical consequences. I conclude by suggesting that the case of the woodpecker troubles a strand of contemporary scholarship in Science and Technology Studies that argues that biotechnologies are inherently transformatory and that foregrounding entanglement and interspecies relations is ethically generative. Instead, a discursive separation of nature and culture may be innovative.

On September 24th, 2002, Mike Webster – a hall of fame American Football player who won four Super Bowls as part of a Pittsburgh Steelers team widely agreed to be amongst the best ever to play the sport – died, aged 50, after suffering a heart attack. As described in *League of Denial*, a book which chronicles Webster’s life and death, Webster experienced years of physical and mental health problems prior to his passing. Webster was divorced, homeless, and suffered extreme mood swings (Fainaru-Wada and Fainaru, 2013: 49–54). He would wrap his feet in duct tape as a remedy to the constant pain caused by long, bleeding cracks that made it difficult to walk. His teeth were falling out and so he simply superglued them back in (Fainaru-Wada and Fainaru, 2013: 84). His ‘fingers were so mangled, his knuckles so swollen, that he couldn’t hold a pen for very long. To write, he used the same duct tape that held his feet together and wrapped it around his fingers’ (Fainaru-Wada and Fainaru, 2013: 92). Perhaps most shockingly, Webster would self-medicate with a high voltage stun gun that he used to fall asleep.

Despite these very evident injuries, it was actually microscopic brain changes that made Mike Webster famous for a second time. Bennet Omalu, a neuropathologist, conducted Webster’s autopsy and prepared slices of Webster’s brain for examination under a microscope. In his autobiography, Omalu described what he saw:

These slides did not appear like they should belong to a fifty-year-old man. Each slide contained numerous brain cells, yet many had died and disappeared, and many appeared like ghost cells. A large number of the remaining cells appeared shrivelled, as if in the midst of the throes of death. I observed spaces – spongiosis – in the substance of the brain, with shrivelled brain skeleton and skeins of brain scars, like a partially demolished building stripped of its windows and its aesthetics gone, leaving behind just the main frames, pillars, and broken-down walls. (Omalu, 2017: 140)

On the basis of these observations, Omalu authored an article that diagnosed Webster as the first football player with Chronic Traumatic Encephalopathy, or CTE (Omalu et al., 2005).

CTE is ‘a neuropathologically distinct slowly progressive tauopathy with a clear environmental etiology’ (McKee et al., 2009: 709), chiefly, traumatic brain injury (TBI). And while it is the neurodegenerative and neuroanatomically distinct tauopathy that defines CTE (McKee et al., 2016), it is the ‘clear environmental aetiology’ which is responsible for much of the attention the diagnosis receives. The people most frequently associated with CTE play contact sports. Those who have been exposed to blast zones in the military as well as others with high rates of TBI, such as victims of domestic abuse, are also understood as being at risk (Casper and O’Donnell, 2020; Stern et al., 2013: 1122). Omalu insists that what he saw in Webster was an entirely new disease entity (Omalu, 2008: 15, 2017: 148, 2020: para. 5). Others (Bachynski, 2019; Casper, 2018a, 2018b) trace a much longer history of ‘punch drunk syndrome’ and ‘dementia pugilistica’ which stretches back at least a century. Regardless, and sociologically, since the turn of the 21st century it is widely argued that there has been a ‘cultural awakening’ (Anderson and Kian, 2012: 156) over the long-term risks associated with TBI. This ‘cultural awakening’ is largely agreed to trace back to Webster and has led to a much discussed ‘concussion crisis’ in sports (Carroll and Rosner, 2012; Nowinski, 2007; See: Malcolm, 2020 for a discussion of the composition, contours, conceptualisation of this ‘crisis’) which is radically reshaping sporting practices and athlete identities (Ventresca, 2019).^1^

In this article, I examine one particularly surprising aspect of the recent controversy surrounding CTE: The highly contested deployment of the woodpecker as an animal model. This is an animal model that is particularly lively outside of the laboratory (see Asdal, 2008: 911), shaping popular narratives of the disease through its outsized presence in leading popular texts as well as acting as an inspiration and marketing tool for emerging biomimetic technologies.

I investigate the role of the woodpecker in four steps. First, I detail Ig Nobel prize-winning research that asks why woodpeckers ‘don’t get headaches’. Second, I examine the enrolment of the woodpecker within sport’s 21st-century concussion crisis: Here I show that the woodpecker is constructed as contra-human, an animal model that is not like us, and thus part of evidence against contact sport. Third, I consider a biomimetic technology – the *Q-Collar*. The Q-Collar is a ‘U’ shaped device worn around the neck that applies a small amount of pressure to the jugular veins, reducing blood flow from the head, and increasing the blood volume within the inter-cranial space: This is a technology that seeks to mimic the physiology of the woodpecker and thus attempts to render sport safer by bridging the gap between humans and birds, transforming the human brain into a woodpecker’s. Fourth, and finally, I turn to criticisms of the Q-Collar that, once again, explicitly seek to open a chasm between woodpeckers and humans. Throughout, my focus is upon both how woodpeckers are used to scaffold diverse ethico-epistemic arguments about the need, or lack of need, for radical social change and what these arguments might tell us about the ethical potential of nature-culture entanglement.

Following the approach of others who have examined the entanglements of nature and society within a context shaped by both popular culture and market economies (e.g. Haraway, 1997), I undertake this analysis by tracing the relationship between woodpeckers and brain injury across a range of sources. In particular, I draw on scientific research published in a number of fields (including ophthalmology, sports science and neuroscience), on writing produced for general audiences and included in text books, autobiographies and investigative journalism, on a Hollywood film, *Concussion*, that dramatized much of the aforementioned work, the patent applications, products and promotional videos produced by biotech’ start-ups and sporting goods manufacturers, and awards ceremonies that have both lauded and laughed at some of the preceding outputs.

It is a frequent claim – from both academia (e.g. Patricios and Kemp, 2014) and the popular press (e.g. Hoge, 2018; Whitlock, 2015) – that media, commerce and science are hard to parse when it comes to CTE. In producing a collage that traces the relations between brain injury and woodpeckers across diverse outputs I seek to recognize that the ‘lines demarcating commercial culture, basic science, natural history for the citizen, business news, visual arts, personal testimonials, and science policy are very blurry’ in matters of biotechnology (Haraway, 1997: 107–108) and that ‘matter comes to matter’ (Barad, 2003) in spaces that far exceed the walls of the laboratory. In order to aid this analysis, in the following sections I foreground two existing areas of study: Nelson’s (2013, 2018) work on ‘epistemological scaffolding’, which I later reconsider in the current context as ‘ethico-epistemic scaffolding’, and recent work on biomimicry. I take this second body of work as being indictive of a certain valorization of the ethical virtue of nature-culture entanglements, a valorization that I argue is problematized in the current case.

## Epistemological scaffolds

In her work on the use of animal models within a pharmacology laboratory, Nelson (2013, 2018) develops the concept of an ‘epistemological scaffold’. Nelson argues that an epistemological scaffold ‘function[s] as a support structure and platform for doing work … a transient structure that can be modified, reconfigured, and adjusted to different heights’ (Nelson, 2013: 7). This ‘scaffold work’ (Nelson, 2018: 85) is conducted in order to support a more enduring and generalizable permanent structure in the form of scientific knowledge and practice.

A case that Nelson details at length involves the use of mice to model human anxiety disorders. The use of mice in this context is non-obvious (do mice suffer from anxiety? Is that anxiety anything like a human’s?) and thus requires significant scaffolding. Nelson states that in order to:

argue for the use of models as an appropriate tool, researchers select particular facts or observations about both the model organism (the mouse) and the organism being modeled (the human) and attempt to link them together, and these paired pieces of information are stacked to build the epistemic scaffold to greater heights. (Nelson, 2013: 8)

Nelson pays particular attention to an experimental set-up known as the ‘elevated plus maze’. The utility of the maze is premised on two more-or-less independent arguments that form the base of the scaffold: An ecological argument (that mice will tend to avoid the open arms of the maze because they are afraid/anxious about heights) and a pharmacological argument (that some delivered drug will increase/decrease anxiety-like behaviours). Some elements of this scaffold are relatively secure (e.g. that the mice will avoid the open arms of the maze when given caffeine), while ‘higher’ levels – that this avoidance is somehow akin to human anxiety, is influenced by genetics, is altered predictably by various classes of drugs, and so forth – are more precarious. Nelson describes scientists who are cautious in conducting ‘scaffold work’ at greater heights and yet must traverse these heights in order to demonstrate the utility of their research to the pressing issue of alleviating anxiety disorders in humans.

In this article, I argue for the utility of understanding woodpeckers as a highly contested part of an epistemological scaffold for CTE. Unlike Nelson, however, I argue that ethics is also central to this picture: Woodpeckers are used to scaffold *both* epistemological and ethical positions. As Nelson’s allusion to building and construction implies, ‘scaffolding’ is intended to enable further types of action: They are a ‘platform for doing work’. In Nelson’s case, the work being undertaken is, primarily, further laboratory work aimed at producing yet more knowledge. In the case of the woodpecker, however, things are more varied. While there is certainly some laboratory research into woodpeckers and brain injury, the type of action facilitated through scaffolding is frequently oriented toward more straightforwardly cultural work: The abandonment of football or, alternatively, the retail of a biomimetic technology that makes the sport safer and enables its continuation. Thus, the work being scaffolded by the woodpecker is both overtly epistemic and overtly ethical in its nature in that it makes claims to knowledge (e.g. the nature of human and woodpecker brains) and proposes a normative course of action (e.g. to play or abandon football).

Paxson and Helmreich (2014) discuss models that are ‘moral exemplars – models that are not simply descriptive, but that might simultaneously be prescriptive’ (p. 171). More generally, ‘is’ and ‘ought’ are consistently collapsed when it comes to ‘nature’; we consistently ‘look to nature as a source of norms for human conduct’ (Daston, 2019: 3). I argue that woodpeckers are deployed within epistemological scaffolds in part because they are ‘persuasive objects’ (Roosth, 2017: 175) or ‘promising tokens’ (Paxson and Helmreich, 2014: 183) that not only embody a description of the world but also compel a particular course of action in order to reimagine how a future world could or should look. This argument concerning the entanglement of knowledge and ethics also speaks directly to bodies of thought in feminist technoscience and new materialism which have highlighted the ethical potential of nature-culture becomings.

## Nature-culture entanglements and biomimicry

Michael (2003) has suggested that STS scholars tend to equate novel, exotic technologies with the production of new social worlds and mundane, existing technologies with the reproduction of existing social worlds. This is particularly evident in technologies that are articulated as being entangled with nature. As Michael (2003: 132) observes, considering scholars of biotechnology such as Conrad, Rabinow and Rose, ‘the exotic is about change, transformation, production, becoming, the mundane is about homeostasis, reproduction and being’. Helmreich, considering much the same group of scholars, argues that despite all their differences ‘definitions of biocapital centre (with varying emphasis) on two *transformations*: in biotic substance and in economic speculation and sentiment’ (Helmreich, 2008: 463 emphasis added; see Birch and Tyfield, 2013: 300–301 for a similar argument). In this body of work we see the possibility of new worlds through novel practices of nature-culture entanglement.

An example of nature-culture entanglement of particular relevance to the current case is that of ‘biomimicry’, described by Goldstein and Johnson (2015: 78) as ‘the practical engineering of inspirational forms found in the nonhuman world’. Goldstein and Johnson report that biomimetic technologies have been embraced within certain strands of the social sciences ‘where an analysis of deeply entangled networks similarly highlights the poverty of the nature-society divide constituted by Enlightenment thought’ (p. 67; also Johnson, 2020; Michael, 2003).

That theoretical embrace of biomimicry is evident, for example, in Barad’s discussion of the topic. Barad states that:

the enterprise of bioengineering is making it crystal clear that the nature-culture dualism is a construction, a point that feminists and other social critics have been trying to get across for some time. (Barad, 2007: 369)

Barad (2007) stresses that this orientation is not ‘technophilic’ (p. 469 fn. 31) and makes clear that there are ‘grave dangers’ (p. 369) associated with biomimetic technology. Still, the claim that biomimetic practices should be ‘the beginning point for ethical considerations’ (Barad, 2007: 369) is illustrative of the stated affinity between biomimetic science and her own commitment to ethico-onto-epistemological entanglement.

Fisch provides a further, considered example of the ethical horizons which are understood to be opened up through biomimetic practice. Fisch sees as problematic those projects that attempt to straightforwardly *mimic* nature, stating that such work ironically reinforces nature-culture dualisms while reserving ‘for itself the role of nature’s interpreter, developing what it declares is the authentic reading of the nature’s underlying principles’ (Fisch, 2017: 817). The work of Janine Benyus and ‘mainstream biomimicry’ (Fisch, 2017: 799) – for example the modelling of the nose of ‘bullet trains’ on the beak of a kingfisher – are exemplars in this regard. By contrast, Fisch sees genuine hope for a progressive, post-colonial and post-human politics in projects *inspired* by nature, noting that ‘[o]riginal, reproduction, authentic and imitation are terms that have no specific meaning in relation to inspiration’ (Fisch, 2017: 818). What occurs instead is more creative and performative.^2^ Such claims resonate with both Roy’s ‘risky argument’ that emerging biotechnologies ‘may be used to dismantle the grid of representation and move us towards a playful proliferation of differences’ (Roy, 2018: 133) and the tendency within strands of contemporary scholarship to engage in an ethical valorization of relationality and entanglement *tout court* (Giraud, 2019: 122).

The assumed promise of biomimicry come despite the widespread deployment of biomimetic technologies in situations that might give ethical pause. ‘[T]he US Department of Defense (DoD)’, for example, ‘has played a much more significant role in the field’s legitimation than any environmental or design movement’ (Johnson, 2020: para. 5), a finding seemingly reaffirmed across a number of empirical sites (e.g. Masco, 2006: 322; Myers, 2015: 25–26). Detailing this relationship between biomimetic science and the military, Johnson argues that:

forms of biological life matter to the US military because of their ability to perform what humans cannot, to dominate an environment in which our bodies have difficulty functioning… the DoD seems to have taken up a call to “become animal” by engaging in a bioinspired turn toward technological innovation. (Johnson, 2015: 308)

The entanglements between the DoD and biomimetic science do not *a priori* foreclose the possibility of ethical transformation – both Barad and Fisch, for example, comment on such entanglements. Furthermore, researchers such as Johnson, who are more critical of the ethical potential of biomimetic science, do not presuppose that nothing changes with the introduction of biomimetic technology; there may in fact be fairly significant ‘mutations’ (Masco, 2006: 301). Nonetheless, there is a clear suggestion that biomimicry and nature-culture entanglements are frequently deployed to aid in the continuation and expansion of existing social and military orders, rather than the emergence of something radically new.

In this article, I contribute to this discussion of the ethical potential of nature-culture entanglement by examining the various mobilizations of the woodpecker in the context of CTE and American Football. The woodpecker is repeatedly deployed as part of ethico-epistemic scaffolds intended to compel diverse courses of action – to save or, alternatively, end football. I find that, in this instance, it is those advancing arguments premised upon the entanglement of woodpeckers and humans via biomimetic technology who promise the continuation of current economic and social practice. Those keeping the woodpecker and the human apart, meanwhile, argue for societal transformation. In the wake of these findings, I argue that more attention needs to be paid to the particular dynamics of a situation before adjudicating on the ethical potential of a given nature-culture entanglement (van Dooren, 2016).

## Analysis

### Why don’t woodpeckers get headaches?

Around the same time as Mike Webster’s death in 2002, Ivan Schwab, an ophthalmologist based at The University of California at Davis, published an article entitled ‘cure for a headache’ that graced the pages of the *British Journal of Ophthalmology* (Schwab, 2002). As editor of the journal, Schwab was also able to ensure that a picture of the bird appeared on the issue’s front cover (Nadis, 2006: 616). In the article, as well as in his later textbook *Evolution’s Witness*, Schwab (2012) addresses the apparently perplexing question of why woodpeckers don’t get headaches. Woodpeckers are tree-dwelling birds found across much of the planet and are perhaps most notable for their eponymous behaviour: drumming on tree trunks in order to feed on insects beneath the bark and communicate with other birds. Focusing on the pileated woodpecker (Dryocopus pileatus) found in North America, Schwab observes that the bird:

may strike the hard surface of a tree at a rate of up to twenty times a second (not a misprint) and up to 12,000 times a day, with deceleration forces of up to 1200 grams with each impact. That’s the equivalent to striking a wall at 16 miles an hour – face first – each time. (Schwab, 2012: 198)

In both the article and the textbook, Schwab details a number of evolutionary adaptations which mitigate against the potentially problematic repercussions associated with hitting a wall, face first, 12,000 times a day – problems which might include retinal detachment, concussion, headaches and the eyes ‘quite literally popping out of the woodpecker’s head’ (Schwab, 2002: 843).

The woodpecker’s adaptions, suggests Schwab, are numerous and remarkable (pp. 198–199). Amongst others, these adaptations include a subarachnoid space that contains little cerebrospinal fluid and that allows the brain to be ‘tightly packed’ within the skull and, perhaps most spectacularly, a tongue that

passes through the right nostril, between the eyes, divides into two, arches over the superior portion of the skull and around the occiput [back of the head] passing on either side of the neck, coming forward through the lower mandible, and uniting into a single tongue in the oropharyngeal cavity … [in order to] create a curious sling-like structure that probably functions as an isometric shock absorber …. (Schwab, 2002: 843)

These adaptions, and the tongue in particular, are sometimes described as giving the woodpecker a natural ‘safety belt’ for its brain (e.g. Wang et al., 2011: 7).

In 2006, ‘Cure for a headache’ would win Schwab an Ig Nobel award, jointly shared with Phillip May, who had undertaken research into the topic several decades before (e.g. May et al., 1976, 1979), and received while wearing a ‘woodpecker headdress’, that is, a bicycle helmet topped with a yellow beak and large red plume. Ig Nobel prizes are awarded ‘for achievements that first make people LAUGH then make them THINK’ (Ig Nobel Prizes, n.d., emphasis in original) and, to this end, it is perhaps noteworthy that an article in *Nature* discussing Schwab’s win describes awardees as ‘embarrassed’ (Nadis, 2006 617), while a piece in *The Telegraph* lists Schwab’s study in an article entitled ‘Pointless research: Top 10 Ig Nobel award winners for silly science’ (Chivers, 2009). Significantly, the general merriment at the Ig Nobels is matched by the jovial tone in Schwab’s article and all of which suggests that, circa 2002–2006, his research into woodpeckers was deemed by all involved to be little more than a curiosity, something to induce a laugh only to be followed, possibly, and if we’re generous, by a moment’s reflection on the wonder of evolution.

Regardless of its intention, Schwab’s work and award appears to have given knowledge of the woodpecker’s resilience to headaches something of a boost. Alongside significant media coverage, citations to both his own and May’s work from the 1970s increased significantly,^3^ as did an emergent academic field looking into the matter. A book for birdwatchers entitled *Why Don’t Woodpeckers Get Headaches* was released in 2007 (O’Connor, 2007) and woodpeckers’ special abilities even got a mention in the 2019 *Lion King* remake.^4^ What was absent from these wide-ranging discussions in the wake of Schwab’s work, however, was any mention of the ‘concussion crisis’ which by 2006 was engulfing American Football in the wake of Mike Webster’s death. The brain damage of Mike Webster and the apparent lack of brain damage in the head-banging woodpecker were left unconnected.

### Woodpecker brains and human remains

At the time of Schwab’s Ig Nobel award there were, in fact, occasional moments when it appeared as if knowledge about humans and woodpeckers might be drawn together. Steve Nadis, writing in *Nature*, says that like ‘boxers, woodpeckers tense their neck muscles before absorbing a blow’ (Nadis, 2006: 616). In other words, humans and woodpeckers may be *alike* in being able to mitigate against acute head injury. Several years later, however, quite a different message was taken from the case of the woodpecker – perhaps most notably through the 2015 film *Concussion* (Landesman, 2015).

Starring Will Smith as Omalu; David Morse as Webster; and Alec Baldwin as Julian Bailes, a well-known neurosurgeon and former doctor to the Pittsburgh Steelers, *Concussion* is a fictionalized retelling of the story underpinning the introduction this essay – Omalu’s (re)discovery of CTE. The film occupies a central place in ongoing discourse about CTE: It has prompted articles in *The Lancet Neurology* (Smith and Stewart, 2016), while social scientists have argued that it has ‘solidified star status for the fragile brain’ (Martin and McMillan, 2020: 2). Alongside *League of Denial*, the film is understood as one of the ‘key media texts’ to have ‘crucially shaped’ the ‘underlying tone of media coverage about CTE’ (Ventresca, 2019: 143). Gallup analysts have gone so far as to suggest that the film may be responsible for a slight decrease in the popularity of the NFL (Sandel, 2020: 170). At the centre of *Concussion* is the assertion that the NFL knew that football caused brain damage and, fearing the economic consequences of CTE, actively attempted to cover up Omalu’s findings in a manner akin to the tobacco industry’s cover-up of the dangers of smoking.^5^

*Concussion* features woodpeckers as a reoccurring motif, moving them into an earnest position at the very centre of Webster’s self-evidently serious story. Around twenty minutes into the film there is a tense meeting between Bailes and Webster. Morse plays Webster as distressed and agitated throughout this meeting. The pair discuss Webster’s medication and Webster pleads with Bailes for help, asking the doctor to ‘fix this’, while grabbing his head. Bailes eases Webster into a seat, giving him a shot of the antipsychotic drug Haldol as he does so. Webster receives this injection from Bailes in the background of the shot while, sitting on Bailes’ desk, in the foreground and for 5 seconds, is a woodpecker skull complete with the safety belt tongue.

While the presence of the skull is not remarked upon in this scene, later, after Webster’s death and the posthumous diagnosis by Omalu, the bird is explicitly drawn upon. In this scene, Omalu tries to explain to his colleagues how football could have damaged Webster’s brain. He reaches into his briefcase for a manilla folder that, upon opening, reveals some pictures of animals. Omalu explains the pictures to his colleagues:

The Cape Gannet, a diving bird capable of generating speeds of up to 75 miles per hour, turning itself into a missile as it collides into the face of the sea. The Red Head Woodpecker can absorb a g-force of 1000, pecking a tree 12,000 times a day, 85 million times over its lifetime. Big Horn Sheep can generate… [Omalu is cut off at this point by his understandably frustrated colleagues]. Okay, okay. All of these animals have shock absorbers built into their bodies. The woodpecker’s tongue extends through the back of the mouth, out of the nostril, encircling the entire cranium. It is the anatomical equivalent of a *safety belt* for its brain. Human beings, not a single piece of our anatomy protects us from those types of collisions. A human being will get concussed at 60 Gs. A *common* head-to-head contact on a football field? 100 Gs. God did not intend for us to play football. (Landesman, 2015, emphasis in original)

The language is strikingly similar – the 12,000 hits a day, the anatomical description of the safety-belt tongue – to that of Schwab during his more light-hearted discussions of the woodpecker.

Both of these scenes from *Concussion* are, in some respects, tethered to non-fiction. The preface to Omalu’s (2017) autobiography, *Truth Doesn’t Have a Side*, is entitled ‘God did not intend for human beings to play football’ and in that preface Omalu (2017) states ‘Woodpeckers can play football safely. Humans cannot …’ (p. 14). *League of Denial* also suggests that Bailes did indeed display ‘a woodpecker skull in a jar on top of his desk. … Every once in a while, someone ask Bailes about the curious object. … He would pick up the tiny bird brain … and explain that if only NFL players were built like woodpeckers, none of this would have happened’ (Fainaru-Wada and Fainaru, 2013: 2).

It is evident from *Concussion* that woodpeckers are being positioned to have an important rhetorical function in the story of CTE. Indeed, and while this work is going on at quite some distance from the laboratory (Asdal, 2008), I suggest that the woodpecker is being introduced to scaffold a particular understanding of CTE and that this scaffold is intended to support both an epistemological and an ethical argument.

Unlike the mice examined by Nelson, where modelling was based upon a form of ‘transposition’ (Friese and Clarke, 2012) wherein the bodies of mice and humans are understood to be in important respects analogous, woodpeckers become entangled with the narrative of Webster’s CTE while, materially, remaining distant from it. If, as Phillip May said in the 1970s, woodpeckers are an ‘experiment in Nature’ (May et al., 1976: 454), then I suggest that they are deployed in order to demonstrate three matters simultaneously.

First, human anatomy is understood here to be of a profoundly, irrevocably different nature to that of the woodpeckers. These are two species who resolutely do *not* meet. Daston has identified one primary use of the word ‘nature’ to be what she calls ‘specific natures’ – the traits ‘which makes it [an organism] what it is and not something else, its ontological calling card’ (Daston, 2019: 7). The demarcation of ‘species’ as part of taxonomic ordering is a primary science of ‘specific natures’, according to Daston, with the label denoting that organisms of different species necessarily have different specific natures. Given that they are evidently different species, it is perhaps unsurprising that woodpeckers and humans are articulated here as radically discontinuous from each other. Nonetheless, and as Friese and Clarke (2012) discuss, acts of generalization and standardization across species boundaries necessarily lie at the heart of work which uses animal models as part of their epistemic scaffolding (see also: García-Sancho and Myelnikov, 2019). The woodpecker, by contrast, is introduced to play a quite different role: they demonstrate not that a trait is generalizable across species, but precisely the opposite. The specific nature of the woodpecker includes ‘shock absorbers’, unlike that of the human.

Second, football is profoundly unnatural, at least for humans. A second use of ‘nature’, according to Daston, is ‘local nature’ – ‘a harmony between people, climates, topographies, and laws … the elements of each formed a harmonious (and sometimes precarious) whole, poised in delicate equilibrium’ (Daston, 2019: 16, 19). Certain species, then, belong in certain spaces and as part of certain ecologies. When Omalu (2017) tells us that ‘[w]oodpeckers can play football safely. Humans cannot’ (p. 14) and Bailes laments that ‘if only NFL players were built like woodpeckers, none of this would have happened’ (Fainaru-Wada and Fainaru, 2013: 2) they draw attention to this point. For these protagonists, it is not that the ecology of the football field *per se* is pathological, it is that humans are in a state of disequilibrium (Daston, 2019: 19) while occupying it. Juxtaposing humans with woodpeckers, who remain in a state of equilibrium within their own head-banging ecological setting, demonstrates this point.

The third rhetorical usage of woodpeckers in the above passages most clearly demonstrates that the scaffold work being performed by woodpeckers is best understood as ethico-epistemic in nature. The prologue to *League of Denial* – the keystone cultural text, alongside *Concussion*, about the discovery of CTE (Furness, 2016; Ventresca, 2019: 143) – is entitled ‘bird brains’, a term that also appears in medic Elizabeth Sandel’s recent book *Shaken Brain* (Sandel, 2020: 17). ‘Bird brain’ is a phrase for stupidity which has been widely used since at least the 19th century (Emery, 2016: 17) and its use in *League of Denial* as an introductory framing device makes explicit a dimension of the discussion which otherwise remains unsaid but lurks in the background throughout: Woodpeckers demonstrate just how ludicrous it is to think that the human body would ever be able to tolerate the kind of forces involved in playing a game like football. The disequilibria caused by a human on a football field should be *obvious*. ‘God did not intend’ for us to be in this space, Omalu states, so organizations like the NFL put players like Webster at risk against the will of God. Leading cultural texts, including those produced by scientists like Omalu, therefore seek to deploy the woodpecker as a ‘persuasive object’ that weaves together epistemological and ethical arguments that both exemplify the costs of football on the human brain and seek to ‘convince and compel’ a future course of action (Roosth, 2017: 175–176).

The centrality of American football to the lives and livelihoods of so many means that suggesting American football needs to be radically overhauled, maybe even banned,^6^ is a claim likely to be vigorously contested. Indeed, Bachynski (2019) has argued that even well into the 2010’s ‘[f]undamentally altering, let alone eliminating, the sport of football was largely not part of the framework advanced by those seeking policy changes to improve sports safety’ (p. 200) while research suggests communities most invested in football are slower to pass concussion safety laws (Rotolo and Lengefeld, 2020). I suggest here that key texts, such as *League of Denial* and *Concussion*, which can be seen as advocating more radical change, scaffold that ethico-epistemic argument, in part, through the radical alterity of the woodpecker.

### Nature tells us there is a solution

‘While human anatomy clearly differs from that of the woodpecker’, states an article from 2012, ‘some correlates do exist in the prevention of TBI’ (Turner et al., 2012: 1111). There is already a hint in this passage that the author of these words sees not an abyssal chasm between woodpeckers and humans, but bridges waiting to be crossed. As did Steve Nadis in the aforementioned *Nature* report on the Ig Nobels, Turner et al. turn to boxing to elucidate these possibilities:

Professional boxers are capable of sustaining forces of great magnitude when preparing for the impact. … Much of this protection has been attributed to a tightening of the neck muscles, one of which is the omohyoid muscle. (Turner et al., 2012: 1111)

Despite this shared ability to tighten the omohyoid muscle, at least two problems remain. First, prior to, say, a blindside hit on a football field, few of us humans are able to prepare for impact in the manner of a boxer (or, indeed, a woodpecker). Second, and the near century long articulation of ‘punch drunk syndrome’ attests to this fact (Casper, 2018a: 10), boxers *do* still get headaches and much worse besides. The capacity of humans to withstand head trauma, even when prepared, is evidently a long way from sufficient.

In a promotional video released some years later, Smith, an author on the 2012 article, explains what he sees as the fundamental problems with human physiology:

When the brain is able to move it is similar to being inside of a car and having your seatbelts not tightened. In traumatic brain injury the brain is able to actually move within its cranial space. We oftentimes refer to this as *slosh*. (Q Collar Canada, n.d.)

As did Omalu, Wang and others, Smith again here turns to the notion of a ‘seatbelt’ – absent, it seems, in humans – in order to understand brain injury. Smith gives this information via a talking-head interview before the screen cuts to the head and shoulders of a computer-generated skeleton with a brain banging around inside the skull. Over the image is written ‘Slosh (släSH) noun. The movement of the brain in the cerebral spinal fluid of the cranium’. This term ‘slosh’, far from being a dictionary definition, was first applied to brain movement by Smith himself, taking it from NASA’s studies of rocket propellants (Turner et al., 2012: 1111).

If the ability to ‘tighten’ the hyoid muscle is thus a point of similarity between woodpeckers and humans, then the fact that the woodpecker’s tongue and hyoid bone constitute a ‘muscular sling’ – an effective safety belt – is an obvious point of difference. It is the goal of the authors to overcome that difference. Smith et al. (2012) suggest that:

Because of the direct proximity of the omohyoid atop of the IJVs [Internal Jugular Veins], it is intriguing to speculate that, on contraction of the omohyoid, perhaps with each peck, the IJVs may be partially occluded and intracranial compliance exhausted (p. 745)

In other words, the woodpecker’s omohyoid muscle ‘may’ partially obstruct the jugular vein, reducing blood flow from the head, increasing the volume of blood within the intracranial space, and thus reducing ‘slosh’ and subsequent brain trauma. However, whereas Omalu – in both the laboratory and in Hollywood – uses this physiological difference to construct an ethico-epistemic scaffold which definitively divided humans and woodpeckers, these authors see a possibility for mimicry and entanglement. The stated goal of the research group is to artificially induce physiological changes found in the woodpecker in other species, and they are intent on ‘biology-inspired discovery’ (Smith et al., 2012: 744) in relation to brain trauma.

One of the articles published in 2012 (Smith et al., 2012: 745) shows a white lab rat, hanging onto a beam and wearing a ‘jugular vein compression device’, a blue collar which fits like a choker necklace. The intent of this collar is ultimately to reduce brain injuries in the rats by narrowing the great divide with woodpeckers. The collar seeks to mimic an aspect of the woodpecker’s physiology discussed above: By applying extra pressure on the jugular vein, it is hoped that the collar reduces slosh in the rat brain, mimicking the neurophysiology of the woodpecker, and affording the animal some protection against head injury.

The authors fitted a small number of lab rats with collars while a 450-gram brass weight was dropped onto prone animals, half of whom had been fitted with the jugular compression collar. When the rats were killed a week later and examined for axonal brain injury, the animals wearing collars were found to have significantly fewer amyloid precursor proteins – implicated in dominant theories of Alzheimer-like dementias (Lock, 2013: 65) – in their brains (Smith et al., 2012; Turner et al., 2012). These rats, as surrogate humans, made to mimic woodpeckers, offered the possibility of reducing brain injury in those liable to suffer brain trauma.

Over the following ten years, up to and including the present moment, the research group would publish dozens of articles, patents and publicity materials associated with the slosh theory of concussion and the collar as a mode of mitigation. Smith had co-founded ‘TBI Innovations’ in 2011 (the conflict of interest is noted on both 2012 publications) and in time a collar based upon that trialled in the rats would be sold, first by *Bauer Hockey* under the name ‘Neuroshield’ and then by *Q30 Innovations* under the name ‘Q-Collar’. This collar would go on to win industry prizes – The *Industrial Designers Society of America* awarded the Q-Collar a ‘gold’ award in their ‘Sport, leisure and recreation’ category, stating: A ‘revolutionary approach to protecting the brain, Q-Collar addresses the problem from the inside out by mimicking the natural defense used by woodpeckers’ (Industrial Designers Society of America, 2017). The collar was made available to purchase in Canada in 2019, the United States in 2021, and with the promise of imminent expansion into Europe. The collar currently retails at C$249/US$199 (approximately €170; £145 at time of writing) and has been worn by both amateur and professional athletes, including a number of players in the NFL (Person, 2018). The Q-Collar, then, is a biomimetic object which has captured a degree of media, industry, scientific and sporting attention. And across these diverse sites the woodpecker scaffolds the Q-collar as an origin story, a bio-mimetic inspiration, a marking tool, an award rationale and as a hook in the popular press.

### The animals in the market

Getting the Q-Collar to market required the enrolment of a huge number of animals variously related to humans as synonym or antonym, sibling or separate, worthy of saviour or worthy of sacrifice.^7^ The efficacy of the collar would be tested on pigs who, like the rats, similarly suffered experimentally-induced brain trauma while fitted with collars and, again like the rats, demonstrated reduced evidence of neuropathological lesions (Mannix et al., 2020; Sindelar et al., 2017). Like Nelson’s rats, these are sacrificial animals (Lynch, 1988) understood as being, in important ways, like humans and thus able to stand in as surrogates.

Quite differently, patent applications from across the time period (e.g. Smith et al., 2014b: 25, 2019: 22) suggest that the Q-Collar might be fitted to protect certain dogs, which are also assumed to be like humans but evidently have different ethical standings than pigs and rats. Noting that smell is often compromised after a brain injury, the patent applicants argue that concussive

injury to Breecher dogs (e.g. bomb sniffers) can be catastrophic. Breecher dogs are inherently exposed to the risk of concussive events and their primary purpose is to help soldiers avoid such an event. Preventing or reducing the likelihood of TBI and associated loss of smell can be critical to the Breecher dog’s mission. (Smith et al., 2014b: 25)

While it is evident that the primary market envisioned for the Q-Collar is the sports market, the possibility that collars might be fitted to both humans and nonhumans at war is thus given serious consideration. Such consideration seems logical given that, and as noted earlier, the military are the most significant funders of biomimetic research and also fund research into concussion and football.^8^ The specifics of the Q-Collar differ from examples which underpin much existing scholarship on biomimicry – such as the ‘robo-lobster’ (Johnson, 2020) or ‘artificial dragonfly’ (Masco, 2006: 322) – inasmuch as the Q-Collar acts to append, rather than replace, human and nonhuman actors in the battlefield. What the Q-Collar has in common with the above examples, though, is its envisaged enrolment within existing institutions and practice.

Despite ongoing concern with dogs and pigs, it is those species that hold biomimetic potential that sustain the most attention. The ‘head ramming sheep’, mentioned in the film *Concussion* alongside the woodpecker, for example, makes a reappearance. Writing in the *New York Times* in an article called ‘Can animals help limit concussions?’, Myer understands the sheep as an animal that can self-induce physiological effects to protect itself from concussion, stating that

the sheep has hollow pneumatic horn cores attached to its respiratory system that allow it to re-breathe its air and thus increase carbon dioxide in its bloodstream, expanding its intracranial vascular tree and enhancing the Bubble Wrap effect. (Myer, 2014)

In an academic context, Myer et al. (2014) re-assert this claim, positioning the sheep alongside the woodpecker as offering a biomimetic solution to brain injury (p. 165). A number of patent applications also spend a significant amount of time discussing the capacity of CO_2_ to modulate the degree of slosh (e.g. Smith, 2018: 4–6; Smith et al., 2014a: 25), taking their lead from sheep physiology. Despite this, interviews with Smith suggest that artificially increasing levels of CO_2_ have been discarded as a biomimetic possibility (Wheeler, 2017).

The woodpecker, meanwhile, is discussed in nearly every patent application, including the most recent (e.g. Smith et al., 2019). As is the case in some of the published literature (e.g. Smith et al., 2012: 745), the bird is marshalled somewhat flexibly in these patent applications, with the biology purportedly mimicked shifting slightly – perhaps an inspiration rather than original (Fisch, 2017). Rather than an exclusive focus on the possible contraction of the omohyoid muscle in order to increase intracranial pressure, the authors on occasion – and as far back as at least 2014 (e.g. Smith et al., 2014a) – state that ‘it is known that the woodpecker has a “pectin [*sic*] apparatus” that protects the globe of the eyeball from the 1200 G impact of pecking’ (Smith et al., 2019: 27). This phrasing suggests that it is the woodpecker’s mode of *eye* protection being mimicked. In the very next paragraph, though, the authors return to noting that the device sold as the Q-Collar ‘raises intracranial volume and pressure and/or intraocular pressure’ (Smith et al., 2019: 27) and published research continues to emphasise that woodpeckers may increase their intra*cranial* pressure/volume via the jugular vein (e.g. Myer et al., 2016: 2). Across sources, it is the capacity of the woodpecker to modulate pressure/volume which is mimicked.

Woodpeckers are, however, rarely mentioned in scientific publications since 2016. These are publications that seek to demonstrate the utility of the collar for reducing neurotrauma, not only in rats and pigs, but in humans acting in settings ranging from military drills (Bonnette et al., 2018; Yuan et al., 2019); to American football (Myer et al., 2016; Yuan et al., 2017, 2018a); to soccer (Myer et al., 2019; Yuan et al., 2018b). Nelson suggests that, once work is complete, the ‘scaffold is dismantled’ (Nelson, 2013: 7) and evidence of its presence disappears. The reduced visibility of the woodpecker within scientific publications can be read in such terms.

In publicly facing material, though, the woodpecker remained strikingly present after the Q-Collar was re-launched by Q30 Innovations in 2019. In a video notable only for its typicality, and after Smith and Julien Bailes^9^ have described the problems caused by brain slosh, a voiceover states that ‘Nature tells us that there is a solution’. The video cuts, with the sound ramped up to a deafened thud, to sheep butting heads and a woodpecker pecking. The camera returns to Smith, who states that:

The head-ramming sheep and the woodpecker; both of these animals have these muscles in their neck called an ‘omo’ which stands for shoulder hyoid muscle and it actually compressed the vasculature of the neck and creates a change in how fast fluids are able to get back out of the brain space.

The image on screen cuts between Smith offering a description of the muscle in question and footage of pileated woodpeckers and rams. A voiceover offers that ‘Now, Q30’s Q-collar brings this breakthrough technology to all sports venues’ (Q Collar Canada, n.d.).

Across patents, publications and publicity materials, the woodpecker is put to work here in ways that are starkly different from the narratives told by those who use woodpeckers to elucidate their horror at the disequilibria of the football field and the damage to players like Mike Webster. While the framing that ‘nature tells us there is a solution’ seems to reinforce the nature-society dichotomy (see: Fisch, 2017), this is at least partially undercut by the insistence on shared physiology in the form of the omohyoid muscle, the jugular vein and the susceptibility to ‘slosh’.

The differences between woodpeckers and humans – so crucial to the ethico-epistemic scaffold of Omalu et al. and detailed above – are overcome biomimetically, shifting the specific nature of the human (and at various points the rat, and the pig, and the dog) so that it becomes one with the woodpecker. This erosion of difference facilitates a process of ‘transposition’ and the ‘back and forth relationships between different lines of work, different spaces and different species’ bodies’ evident in much animal modelling (Friese and Clarke, 2012: 34). By mimicking the physiology of the woodpecker, the human is made to be at home, and in a state of equilibrium, on both the football and the battle-field.

An important consequence follows from this novel articulation of the woodpecker. The ethico-epistemic project of Omalu et al. – premised upon the disentanglement and the radical alterity of humans and woodpeckers – scaffolded a straightforward solution to CTE and the death of players like Mike Webster: Stop playing football. Abandon this cultural practice. The ‘solution’ that Q-Collar offers is evidently quite different: A safer but fundamentally unaltered sport. This is the entire goal of the project. It is the nature-culture hybrid, the entangled biomimetic posthuman, who stands with continuity and the status quo.

### What is it like to be a woodpecker?

This rearticulation of the woodpecker as an epistemological support for the Q-Collar requires a lot of scaffolding. At a minimum, four more-or-less independent scaffolds are essential: (i) The ‘slosh theory’ of concussion that suggests that it is movement within the skull that results in brain damage; (ii) The ‘de-sloshed’ woodpecker that suggests that woodpeckers don’t get headaches because their specific nature (e.g. their seat-belt tongue) ensures that their brain does not slosh; (iii) The symmetry of local natures which suggests that the a woodpecker pecking on a tree and a football player being hit on the field are equivalent in a meaningful way; (iv) The ‘mimicry argument’ which suggests that the collar successfully facilitated the transposition of the woodpecker physiology into the human. This is, undeniably, a very shaky scaffold. While Nelson (2013, 2018) describes scientists who operate at extreme caution at height, they never come close to a site *this* precarious. This difference seems to reaffirm a distinction between Nelson’s mice, models primarily intended to scaffold further laboratory work, and the woodpeckers, intended here to ungird the sale of a sporting good. As an epistemological foundation, the woodpecker may provide little security but, culturally, it continues to compel action. The makers of the Q-Collar have, after all, already taken the technology to market.

Given both the potentially shaky epistemological grounding of the scaffold, the ethically charged nature of brain injury in football, and, in the broader context of CTE, the frequent accusations of conflict of interest, it in unsurprising that, first, there have been consistent and vigorous attempts to pull down the scaffold supporting the Q-Collar and, second, woodpeckers have been central to these attempts. James Smoliga, author of several critiques of the Q-Collar (e.g. Smoliga and Zavorsky, 2017a, 2017b), specifically addresses the issue of woodpeckers in an article with Lizhen Wang, who has written extensively on the biomechanics of woodpeckers (Smoliga and Wang, 2019). Published in the high-impact *British Journal of Sports Medicine*, the short article is a withering attack on ‘the woodpecker model of traumatic brain injury’ (Smoliga, 2018). Smoliga and Wang note that, and as discussed above, woodpeckers have multiple adaptations to protect their brains; woodpecker brains are quite different to human brains; and that the biomechanics of a woodpecker peck are quite unlike the rotational hits experienced on a sports field. The authors here echo Friese and Clarke, who note, drawing on work by Star (1983) that ‘social practices of transposition include “simplification work”’ (Friese and Clarke, 2012: 37) by suggesting that the simplification work behind the Q-Collar elides significant interspecies difference.

Perhaps most damningly, the authors suggest that there is no evidence that ‘woodpeckers contract their omohyoid muscle to occlude the jugular vein during pecking’ (Smoliga and Wang, 2019: 1262), potentially undercutting a biomimetic rationale of the Q-Collar. Writing with Zavorsky, Smoliga largely couches his criticism in terms of disciplinary difference (Smoliga and Zavorsky, 2017a: 756) but with Wang they are more direct, stating that the woodpecker model of TBI was ‘proposed by those without a history of woodpecker research, but with a financial interest’ (Smoliga and Wang, 2019: 1262). By foregrounding the woodpecker in these critiques, there is again an apparent recognition that the woodpecker is a ‘persuasive object’ (Roosth, 2017: 175) at the centre of the ethico-epistemic scaffold of the Q-Collar. If the ‘prescriptive character’ of the woodpecker were ‘undone’ then the whole scaffold may fall (Paxson and Helmreich, 2014: 180).

One striking criticism of the Q-Collar and related biomimetic endeavours is that while the assumption has been that woodpeckers don’t suffer neuropathological damage as a result of their activity, we don’t actually *know* whether this is the case. It is assumed that woodpeckers don’t get headaches but who has asked? To this end, Farah et al. (2018) studied the brains of a variety of woodpeckers procured from museum collections and found that ‘pecking may induce the accumulation of tau in the woodpecker brain’ (Farah et al., 2018: 1). The authors state that while neuroanatomical differences prevent a direct comparison, the ‘anatomic locations and staining patterns of the lesions identified in the brains of woodpeckers share some similarities to human CTE’ (Farah et al., 2018: 9). Unlike other attempts to shake the scaffold which are premised upon a prising reopen of the human-woodpecker divide, Farah and colleagues continue to envisage the lives of humans and woodpeckers as entangled. The interspecies divide, however, is crossed not by making the human more woodpecker, but by making the woodpecker more human. The woodpecker may not, it seems, be quite as home on the football field as we would imagine.

## Discussion

My focus in this essay has been less upon what the Q-Collar *is* – whether it is successfully mimicking the woodpecker, whether the criticisms are valid, whether the technology works and so forth – but on the ‘how, why and what of nature’s authority in the human realm’ (Daston and Vidal, 2004: 1) and the allied question of ‘what counts as nature, for whom, and at what cost’ (Haraway, 1997: 104). Just a few years ago, the woodpecker’s ability to peck without apparent injury was represented by a scientist collecting an Ig Nobel award, wearing both a tuxedo and bicycle helmet with a big yellow beak and enormous red feathers while making jokes about a male woodpecker’s inability to satisfy its partner because of a headache. But the topic came to play a significant role in a debate about biomimetic technology, neurodegenerative disease and the future of ‘America’s game’.

Following this story through various organizations (design companies, scientific establishments, news organizations, football teams, Hollywood studios) and outputs (patent applications, best-selling books and autobiographies, websites, scientific publications, publicity videos) shows that, through the woodpecker, a menagerie of humans, rats, pigs, sheep and bomb-sniffing dogs have been diversely enrolled in projects that have, first, radically challenged the safety of football and, second, led to the development of the ‘Q-Collar’, a biomimetic technology intended to ward against neurodegenerative disease. I conclude by briefly thinking across these projects and reflecting on the ethical importance of these diverse ethico-epistemic scaffolds.

In a video produced by local newspaper *The Columbus Dispatch* (2017) and entitled ‘New device aims to prevent concussions by mimicking makeup of woodpeckers’, a senior biomedical engineer on the Q-Collar project sits at his computer in a chequered shirt and with a coffee to go. He smiles and stresses his excitement at this work. The engineer scrolls through the contents of his computer telling us, first, about the concussion crisis in sport and, second, how woodpeckers offer a possible way out. He concludes by talking about the motivation of those involved in the project:

I know that when Julien [Bailes] talks about this project, his goal is to save football. He loves football. He played football, his kids played football, he wants to save football. Greg [Myers] is a huge football fan, highly involved with the [Cincinnati] Bengals, and wants to save football. So, everybody on this team likes the impact of what this might mean for sports, for keeping youth active, and for protecting the brain. It’s actually very hard for me to watch football, to watch hockey, and not see a device on the neck …. (*The Columbus Dispatch*, 2017)

The keywords here, I think, are not only the reoccurring ‘save’ but also the ‘keep’ and the ‘protect’. A human successfully rendered woodpecker via biomimetic technology is quite explicitly put to work for the status quo: to *save* football.

Recently, Giraud (2019) has asked the question *What Comes After Entanglement?*. A good deal of scholarship has answered Giraud’s question by equating the development of exotic technologies that purport to entangle nature and culture with the production of new social worlds (see: Michael, 2003). This scholarship suggests that forms of entanglement should be ‘the beginning point for ethical considerations’ (Barad, 2007: 369), positing that, while ‘risky’ (Roy, 2018: 133), nature-culture entanglement necessarily holds the possibility of a progressive, post-colonial politics (Fisch, 2017).

Here the conclusion reached is quite different: What comes after entanglement is continuity. Rendering a human-woodpecker through biomimetic intervention and nature-cultural entanglement undercuts the moral authority of nature to demand change. By altering the ‘specific nature’ (Daston, 2019) of the human, the disequilibria of the football field is put right, and the possibility of leaving cultural and capital practices untouched becomes imaginable. Far from a radical break, the entanglement of nature and culture destabilizes the ethico-epistemic scaffold used to promote change and ultimately leads to continuation. On the other hand, those who refuse to bring nature and culture together, who strive to keep the football player and the woodpecker apart, understand themselves as acting in a manner that will lead to an overhaul of culture, *viz*, an American society in which football is banned or altered significantly.

I am not alone in reaching this conclusion in the context of biomimicry: Goldstein and Johnson (2015: 76) argue that ‘biomimicry conscripts a more-than-human world into the business of economic and social development, making life’s continuation entangled with capital’s expansion’. Masco (2006: 322) similarly understands the deployment of biomimetic insects in the New Mexico desert primarily in terms of expansion and continuation, arguing that these activities should be understood as the ‘genealogical descendent of the biological testing programs conducted during above-ground nuclear testing’ as part of The Manhattan Project.

To reach this conclusion that biomimicry can work to re-produce existing social worlds rather than create new ones does not necessitate ‘buy[ing] into a simplistic nature/culture dualism’ (van Dooren, 2016: 43), nor does it suggest that biomimetic entanglement can never have emancipatory potential. Rather, it is a call to be attentive to ‘the particular dynamics of diverse forms of human relationship with specific non-human others’ (van Dooren, 2016: 43), to acknowledge that any given entanglement may itself constitutively exclude the possibility of radical social change (see: Giraud, 2019), and to keep alive the possibility that it may often be forms of withdrawal and separation which offer the possibility of new forms of life (van Dooren, 2016).

Foregrounding the specificity of particular entanglements when considering their ethico-epistemic potential leads to a second conclusion: The ethics of diverse entanglements are both rarely equal and highly differential in their consequences. This conclusion is obscured by a singular focus on a ‘human-woodpecker hybrid’. The dominant hybrid across outputs from Q30 is that of a human rendered woodpecker and able to carry on playing football – but this hybrid is far from the only option offered. As Friese and Clarke (2012) have noted, ‘the increased use of a species in scientific research results in greater knowledge regarding that species’ (p. 42). Here, the ethico-epistemic stakes of woodpecker headaches have led to an increase in research, which raised the possibility that woodpeckers do, in fact, suffer neuropathological damage akin to that suffered by Mike Webster. This, evidently, is a quite different scenario from that in which the woodpecker is rendered human and brought into culture. This second rendering unties the knot binding Q30 Innovations, the woodpecker, and the football player: Certainly, critics believe that such a rendering would count against the Q-Collar. The woodpecker, meanwhile, lies untied but newly pathologized and medicalized, lying on an autopsy table at Boston University, and ready to be deployed as a quite different type of model in the fight against neurodegenerative disease (Farah et al., 2018: 10; Gabbatiss, 2018). The corpse first made us laugh, but then made us think.

## Notes

1. While it is not the focus of this article, it needs to be noted that CTE remains a highly uncertain diagnostic entity. In a recent article, a leading scientist argued that: there are no findings from epidemiological studies to determine the prevalence of CTE in the general population. There are no universally established methods for making the diagnosis on either a neuropathological or clinical basis or for distinguishing features of the disorder from other established conditions, such as Alzheimer’s disease or major depressive disorder. Lastly, while the disease is believed to result from long-term exposure to repetitive subconcussive blows, no success has been obtained in defining the requisite level of impact or in obtaining evidence from basic laboratory studies to establish causality. (Barr, 2020: 155) In a fraught area of study, others (e.g. Casper et al., 2019) argue that this foregrounding of uncertainty is *itself* incredibly problematic, a strategy (see: McGoey, 2012) akin to that found in the lobbying of tobacco companies and climate change denialism.

2. Without disputing the utility, the distinction between ‘mimic’ and ‘inspire’ is largely Fisch’s, rather than a member category. As Fisch notes, key texts in the field elide the difference: The title of Benyus’ touchstone text *Biomimicry: Innovation Inspired by Nature* is an obvious case in point.

3. May et al. (1976, 1979) has two reasonably well cited articles on the topic, both published in the 1970s. According to a Google Scholar search (15th of April, 2020) these publications had been cited, respectfully, 18 and 16 times between publication and 2005. Between 2006, when the Ig Nobel was awarded, and 2019 they have been cited a further 79 and 52 times. Schwab’s article from 2002 was not cited prior to 2006 and has been cited 24 times since.

4. Zazu, a major-domo hornbill states that ‘I had a cousin once who thought he was a woodpecker! He would slam his head … into trees. Our beaks are not built for that. He was regularly concussed!’

5. In the context of CTE more broadly, this comparison to the tobacco industry is frequently made (e.g. Bachynski, 2019: 155; Fainaru-Wada and Fainaru, 2013: 6; Omalu, 2008: 43) although the validity of the comparison continues to be questioned by some (e.g. Barr, 2020: 155).

6. Omalu (2017: 174) is of the opinion that ‘children under the age of eighteen should not be allowed to play football or other high-impact, high-contact sports’ and has suggested that allowing children to play tackle football meets the definition of child abuse (p. 272). He has generally deferred to individual liberty when considering the legality of football in adults.

7. As Haraway makes clear, being understood as a ‘sibling species’ (Haraway, 1997: 113) does little to protect against being rendered ‘killable’ (Haraway, 2008: 80). This is precisely because ‘her suffering promises to relieve our own, she is a scapegoat and a surrogate’ (Haraway, 1997: 47). For more on ‘killability’ see Schrader et al. (2017).

8. See, for example, https://www.ncaa.org/about/resources/media-center/news/ncaa-dod-launch-concussion-study

9. Given Julian Bailes’s prominent role in both the (re)discovery of CTE and the film *Concussion* – he is a central player in both the scenes from *Concussion* discussed above – it is worth noting that Bailes’s intent to save football through the Q-Collar is strikingly divergent from the abolitionist approach advocated by Omalu. The pair do, however, continue to work together as co-directors of a (relatively small) brain bank. That they should continue to work together despite their competing ethical frames is perhaps surprising, although it is far from the only contradiction: For example, Bailes both praises and contributes to, a popular text called *Brainwashed* (Hoge, 2018) wherein a key contributor (Peter Cummings) is a neuropathologist who has argued that, first, woodpecker brains exhibit tau accumulations which share similarities with CTE and, second, that this finding potentially undermines technologies intended to procure safety by mimicking the woodpecker, chiefly the *Q-Collar* (Farah et al., 2018). This contribution is mentioned in *Brainwashed* (Hoge, 2018: 104). It is hard to know how much significance should be attached to these activities which appear to conflict ethically, epistemologically, and, frankly, commercially. They do, though, seem important to note and I thank the anonymous reviewer for making the point.
